# Cell Metabolism Therapy by Small Natural Compounds

**DOI:** 10.3390/ijms241813776

**Published:** 2023-09-07

**Authors:** Salvatore Nesci, Anna Spagnoletta, Francesca Oppedisano

**Affiliations:** 1 Department of Veterinary Medical Sciences, Alma Mater Studiorum—Università di Bologna, 40064 Ozzano Emilia, Italy; salvatore.nesci@unibo.it; 2 ENEA, Italian National Agency for New Technologies, Energy and Sustainable Economic Development, Department of Sustainability, Trisaia Research Center, 75026 Rotondella, Italy; 3 Department of Health Sciences, Institute of Research for Food Safety and Health (IRC-FSH), University “Magna Græcia” of Catanzaro, 88100 Catanzaro, Italy; oppedisanof@libero.it

Cellular metabolism therapy counteracting metabolic dysfunction performs a preeminent role in the pathophysiology of different diseases, such as cancer, diabetes, metabolic syndrome, and cardiovascular and neurodegenerative diseases. The interactions at the molecular level between chemical mediators and cellular systems arise from biochemistry and molecular biology studies that provide the basis of drug therapy [1].

The understanding and study of the mechanisms involving the alteration of bioenergetic metabolism, the overproduction of reactive oxygen and nitrogen species (ROS and RNS), the imbalance between proliferation and cell death, and the interaction between enzymes and substrates might represent a useful and fundamental tool for developing new therapeutic approaches that are more efficient and ensure an improved quality of life.

To this end, the extensive research of putative natural mediators of cellular metabolism, both endogenous and exogenous (derived from animal and plant sources), becomes an intriguing research issue that needs to be expanded upon. The abundance of bioactive chemical compounds discovered in nature, each of which has a potential therapeutic impact on sustainability as well as greater tolerance and bioavailability, is an excellent alternative to synthesised drugs [2].

The ameliorated knowledge about impaired metabolic pathways and the capacity of natural compounds to support and modify them is an important milestone and a significant step forward for science. Given the historical context, it is critical to concentrate the entire scientific community’s attention on the remarkable therapeutic potential of natural compounds, particularly their safety for health.

Mitochondrial dysfunction is the cause of the bioenergetic alteration triggering pathological conditions [3], and excessive ROS production in mitochondria leads to biomolecule damage in the cell responsible for the inflammatory process characterizing different human diseases. Nesci et al. explain that reducing oxidative stress with ROS scavenger bio-compounds counteracts inflammation, highlighting the relationship between inflammation, altered mitochondrial oxidative activity in pathological conditions, and the beneficial effects of phytosomes in one context of non-pharmacological therapy [4].

However, some natural compounds have toxic effects on mitochondria, and PAT (4-hydroxy-4H-furo [3,2c] pyran-2 [6H] -one) is a mycotoxin acting as a competitive inhibitor on the mitochondrial carnitine/acylcarnitine carrier (CAC). By suppressing the β-oxidation following CAC inhibition, PAT causes a consequent decrease of oxidative phosphorylation and ROS production in mitochondria [5].

Novel anticancer and anti-inflammatory phytochemicals are biological properties of Italian *Santolina pinnata*, used in traditional medicine for its valuable source of phytochemicals that trigger apoptotic death in breast cancer cells, decreasing the metastatic ability of tumour cells, and exert anti-inflammatory effects, modulating the cascade mediated by NF-kB in LPS-activated macrophages [6].

Cell metabolism can switch from an oxidative to an anaerobic profile in proliferating cells according to the Warburg effect, increasing glucose metabolism. The novel glycolytic inhibitor gnetin H (GH) directly controls the gene involved in cellular glucose homeostasis via a cytostatic effect in melanoma and glioblastoma cells. Moreover, used in combination with phenformin, a mitochondrial complex I inhibitor, it is a compounds for therapy-resistant tumours, inducing metabolic catastrophe and apoptosis in cancer cells [7]. However, apoptosis-inducing anticancer agents such as doxorubicin (DOX) induced cardiovascular toxicity caused by mitochondrial dysfunction. Bergamot polyphenolic fraction (BPF) can provide beneficial effects on the mitochondrial bioenergetics of porcine aortic endothelial cells (pAECs) treated with DOX. Indeed, the mitochondrial parameters of oxidative metabolism of pAECs impaired by DOX were re-boosted with the use of BPF [8].

Cardiovascular diseases and atherosclerosis are pathological conditions triggered by metabolic disorders such as dyslipidemia. The hypolipidemic potential of a picrocrocin-enriched fraction obtained from the stigmas of *Crocus sativus* L. has been investigated in HepG2 cells by Frattaruolo et al. [9]. The hypolipidemic properties of picrocrocin extracted from saffron stigmas exert a reduction of triglycerides synthesis and an increase in LDL re-uptake through a non-statin-like mechanism.

The nitric oxide (NO) signalling molecule generated by organic nitrates present in the diet is a well-known and effective therapy for cardiovascular disorders. Glyceryl trinitrate (GTN) is a donor of NO that might cause, with the chronic use of this compound, a “nitrate tolerance”. Some natural compounds that could potentially supplement pharmaceutical therapy by providing the NO required to lower GTN intake are explained by Maiuolo et al. [10].

Biocompounds are not used alone to counteract pathological conditions of illness; bioactive molecules such as hyaluronic acid (HA) are also attracting strong interest in various biomedical aspects. Biomedical and pharmaceutical research on HA-based products covers skin benefits to cancer therapy, and from inflammatory disorders to chronic illnesses [11].

On balance, insights and data on the biochemical interactions of known and unknown natural substances with cellular metabolism in health and disease can help to develop new and innovative therapeutic approaches.
